# Gender-specific soluble α-klotho levels as marker of GH deficiency in children: a case–control study

**DOI:** 10.1007/s40618-022-01757-y

**Published:** 2022-03-13

**Authors:** V. Guarnotta, G. Pizzolanti, R. Petrancosta, S. Radellini, C. Baiamonte, C. Giordano

**Affiliations:** Dipartimento di Promozione della Salute, Materno-Infantile, Medicina Interna e Specialistica di Eccellenza “G. D’Alessandro” (PROMISE), Sezione di Malattie Endocrine, del Ricambio e della Nutrizione, Università di Palermo, Palermo, Italy

**Keywords:** Growth hormone, Diabetes mellitus, Insulin resistance, Pediatric, GHD

## Abstract

**Purpose:**

To evaluate circulating soluble α-klotho (sαKL) levels in GHD children before and after 12 months of GH treatment (GHT).

**Methods:**

Auxological and basal metabolic parameters, oral glucose tolerance test for glucose and insulin levels, insulin sensitivity indices and klotho levels were evaluated before and after 12 months of follow-up in 58 GHD children and 56 healthy controls.

**Results:**

At baseline, GHD children showed significantly lower growth velocity standard deviation score (SDS) (*p* < 0.001), bone/chronological age ratio (*p* < 0.001), GH peak and area under the curve (AUC) after arginine test (ARG) (both *p* < 0.001) and glucagon stimulation test (GST) (*p* < 0.001 and 0.048, respectively), IGF-1 (*p* < 0.001), with higher BMI (SDS) (*p* < 0.001), WC (SDS) (*p* = 0.003) and sαKL (*p* < 0.001) than controls. After 12 months of GHT, GHD children showed a significant increase in height (SDS) (*p* < 0.001), growth velocity (SDS) (*p* < 0.001), bone/chronological age ratio (*p* < 0.001) IGF-1 (*p* < 0.001), fasting insulin (*p* < 0.001), Homa-IR (*p* < 0.001) and sαKL (*p* < 0.001) with a concomitant decrease in BMI (SDS) (*p* = 0.002) and WC (SDS) (*p* = 0.038) than baseline. At ROC curve analysis, we identified a sαKL cut-off to discriminate controls and GHD children of 1764.4 pg/mL in females and 1339.4 pg/mL in males.

At multivariate analysis, the independent variables significantly associated with sαKL levels after 12 months of GHT were the oral disposition index (*p* = 0.004, *β* = 0.327) and IGF-1 (*p* = 0.019, *β* = 0.313).

**Conclusions:**

Gender-related sαKL may be used as a marker of GHD combined to GH and IGF-1. Insulin and IGF-1 are independently associated with sαKL values after 12 months of GHT.

## Introduction

Growth hormone deficiency (GHD) affects about 1 out of 4000 children [1]. GHD is characterized by short stature, altered body composition (reduced muscle mass and increased adiposity) [2, 3] as well as metabolic alterations (increase in fat mass) [4–6], and recombinant human (rh) GH treatment (GHT) may result in a reversal of these effects [7, 8].

The diagnosis of GHD is based on many factors including clinical, auxological, and biochemical parameters. As GH is secreted in a pulsatile way, basal GH measurements are useless, and the secretion is assessed using stimulation tests [9].

Children with normal GH secretion and GHD frequently show superimposable peak GH concentrations [9]. Only a minority of children with idiopathic GHD remain GH deficient after discontinuation of GHT [10–13], and about 20% of healthy children may test “deficient” if a single stimulation test is used [14]. For these reasons, two stimulation tests are recommended to assess the diagnosis of GHD [14]. In addition, serum biomarkers (e.g., IGF-1, IGFBP-3) are not fully accurate in distinguishing between GH sufficient and deficient children [15].

A strong relationship between klotho protein and GH/IGF-1 system has been demonstrated [16].

The Klotho gene was first identified in 1997 as an anti-aging agent [17]. An impairment in its genetic expression has been associated with short lifespan, infertility, atherosclerosis, metabolic syndrome, skin atrophy, osteoporosis, and emphysema in mice [17], while over-expression leads to a longer life span [18, 19]. The Klotho gene encodes the alpha klotho (α-klotho) protein composed by an intracellular and transmembrane domain and an extracellular domain [20, 21]. The transmembrane form (mKL) is a co-receptor of fibroblast growth factor 23 (FGF23), which regulates calcium and phosphorus homeostasis [20, 22]. From the cleavage of the extracellular domain mediated by proteases ADAM, can be obtained the soluble form (sαKL), a circulating molecule with endocrine functions [23]. Several effects of sαKL have been reported on insulin physiology, inhibition of insulin/IGF-1 receptor phosphorylation and signalling events, such as tyrosine phosphorylation of insulin receptor substrates and phosphoinositide 3-kinase, thereby blocking insulin and IGF-1 signalling [18, 24].

In the current study, we evaluated sαKL levels in a cohort of GHD children at diagnosis, and during GH treatment (GHT) to assess its potential diagnostic role in GHD.

### Materials and methods

We prospectively studied 58 pre-pubertal children (32 males and 26 females, mean age 7.8 ± 1.7 years) with isolated idiopathic GHD, consecutively admitted to the Endocrinology Section of the University of Palermo during the years 2016–2018 and treated with rhGH for at least 12 months. Fifty-six healthy short children, matched for sex (36 M, 20 F), age (mean age 8.4 ± 1.9 years) and pubertal status, were recruited among patients referred for assessment of short stature as a control group and followed up at baseline and after 12 months. Both controls and GHD children were pre-pubertal during the observation period.

Exclusion criteria were the following: having a multiple pituitary hormone deficiency or panhypopituitarism, not having completed the 12 months of follow-up and pubertal onset before and during treatment. The diagnosis of GHD was assessed according the criteria of the GH Research Society [9]. Both the patients and the controls underwent two stimulation tests (arginine and glucagon) for their short stature or reduced height velocity SDS. Controls had a GH response > 10 μg/L to both stimulation tests.

All patients with GHD received replacement recombinant human GH therapy once daily at bedtime with a pen injection system. Children were treated with an initial mean daily dose of 0.025 mg/kg from baseline until the sixth month and a mean dose of 0.028 mg/kg from months 6 to 12. The decision to change the dose of rhGH therapy was based on the biochemical and auxological parameters, as previously reported [25]. During the entire follow-up IGF-1 levels were maintained within the normal range for age (81.3–255.3 mcg/L for males and 85.9–323 mcg/L for females).

### Study protocol

At baseline and after 12 months of follow-up in controls and GHD children body height, growth velocity, body mass index (BMI), waist circumference (WC) [expressed as Standard Deviation Score (SDS)] and bone/chronological age ratio were evaluated.

The arginine test (ARG) and glucagon stimulation test (GST) were performed at baseline as provocative tests to diagnose GHD and the areas under the curve (AUC) of GH (AUC_GH_) were calculated. GHD was defined when GH peak was < 10 μg/L after two provocative tests. Brain MRI was performed in all children with a GH response < 10 μg/L after the two stimulation tests. No pituitary abnormalities were detected in the group of patients enrolled.

Fasting blood glucose and insulin, hemoglobin A1c (HbA1c), total and high-density lipoprotein (HDL) cholesterol and triglycerides, IGF-1 and sαKL were assayed. Low-density lipoprotein (LDL) cholesterol levels were calculated by the following formula: total cholesterol – (HDL cholesterol − triglycerides/5)*.* We performed an oral glucose tolerance test (OGTT), with 1.75 g/kg body weight of glucose, with blood samples collection every 30 min up to 2 h for glucose and insulin measurements.

The homeostasis model assessment estimate of insulin resistance (Homa-IR) [(fasting glucose × fasting insulin)/22.5] [26], the Matsuda index of insulin sensitivity (ISI-Matsuda) [10,000/glucose (mg/dL) × insulin (mU/mL) × glucose mean × insulin mean] [27], the oral disposition index (DIo) [(ΔInsulin 0–30/ΔGlucose 0–30) × (1/fasting insulin)] [28] and the area under the curve for insulin (AUC_2h insulinemia_) and glucose (AUC_2h glycaemia_) were calculated.

The population evaluated in the current study was different from that enrolled in another study of our group [25].

The study was approved from the Ethics Committee of the Policlinico Paolo Giaccone Hospital, University of Palermo, in agreement with the ethical standards of the local committee on human experimentation (institutional and national) and with the Declaration of Helsinki (1964). At the time of hospitalization, all patients and their parents gave informed written consent to the study and for scientific use of the data.

### Hormone and biochemical assays

Biochemical parameters were measured with standard methods previously reported [25].

Serum samples for sαKL concentration were analyzed using a commercial solid phase sandwich ELISA (Enzyme-linked Immunosorbent Assay) assay kit (cat.27998, Immuno-Biological Laboratoires Co., Fujioka-Shi, Japan). The normal measurement range was 93.75–6000 pg/mL [29]. Samples were assayed following the manufacturer’s instructions.

### Statistical analysis

The Statistical Package for Social Sciences SPSS version 19 was used for data analysis. Baseline characteristics were presented as mean ± standard deviation (SD) for continuous variables, while rates and proportions were calculated for categorical data. Normality of distribution for quantitative variables was assessed with the Kolmogorov–Smirnov test. The differences between the two independent groups (GHD children vs. controls) were evaluated by Student’s *t* test, while the differences between paired continuous variables (before and after 12 months of follow-up in GHD children and controls) were analyzed by the paired *t*-Test. ROC curve analysis was performed to identify the sαKL cut-off differentiating children with GHD from healthy controls.

The independent variables associated with the dependent variable (sαKL) after 12 months of GHT were evaluated by multivariate analysis. A *p* value < 0.05 was considered statistically significant.

## Results

The clinical, hormonal, and metabolic parameters of control subjects and GHD children at diagnosis and after 12 months of GHT are shown in Table 1*.*

**Table 1:** Clinical, hormonal and metabolic parameters of controls and GHD children at diagnosis

	Controls No 56	GHD No 58	*p*
	Subjects (%)	Subjects (%)	
Gender			
Males	36 (64.3%)	32 (55.1%)	0.354
Females	20 (35.7%)	26 (44.9%)	

	Mean ± SD	Mean ± SD	
Age (years)	8.4 ± 2.75	7.8 ± 1.75	0.130
Height (SDS)	− 1.8 ± 0.5	− 2.11 ± 0.71	0.198
BMI (SDS)	− 0.99 ± 0.47	− 0.51 ± 0.27	< 0.001
Waist circumference (SDS)	− 0.3 ± 0.15	0.3 ± 0.1	0.003
Height velocity (SDS)	0.79 ± 0.19	− 0.95 ± 0.53	< 0.001
Bone/chronological age ratio	0.89 ± 0.09	0.76 ± 0.13	< 0.001
GH peak during ARG (µg/L)	15.1 ± 5.91	4.27 ± 3.65	< 0.001
AUC_GH_ during ARG (µg/L)	931.7 ± 397.1	490.2 ± 155.4	< 0.001
GH peak during GST (µg/L)	11.4 ± 4.1	3.55 ± 2.5	< 0.001
AUC_GH_ during GST (µg/L)	598.1 ± 376.3	249.9 ± 158.1	0.048
IGF-I (µg/L)	100.5 ± 12.5	74.1 ± 27	< 0.001
Fasting glucose (mmol/L)	4.11 ± 0.42	4.32 ± 0.59	0.402
Fasting insulin (µU/mL)	4.92 ± 3.10	6.2 ± 3.3	0.380
HbA1c (%)	5.24 ± 0.29	5.2 ± 0.33	0.537
Homa-IR	1.2 ± 0.75	0.87 ± 0.46	0.680
ISI-Matsuda	11.6 ± 4.2	12.2 ± 4.5	0.829
Oral disposition Index	0.56 ± 4.21	0.48 ± 4.47	0.799
Total cholesterol (mmol/L)	3.89 ± 0.52	4.14 ± 0.71	0.694
HDL cholesterol (mmol/L)	1.65 ± 0.33	1.61 ± 0.21	0.320
LDL cholesterol (mmol/L)	1.98 ± 0.56	2.02 ± 0.69	0.839
Triglycerides (mmol/l)	1.61 ± 0.51	1.49 ± 0.54	0.533
sαKL (pg/mL)	1594.5 ± 461.3	1136.5 ± 649.9	0.001

*GHD* GH deficiency, *SDS* standard deviation score, *BMI* body mass index, *WC* waist circumference, *ARG* arginine test, *GST* glucagon stimulation test, *AUC* area under the curve, *Homa-IR* homeostasis model assessment estimate of insulin resistance, *ISI* insulin sensitivity index, *sαKL* soluble α-klotho

At baseline, GHD children showed significantly lower bone/chronological age ratio (*p* < 0.001), GH peak and AUC after ARG (both *p* < 0.001) and GST (*p* < 0.001 and 0.048, respectively), IGF-1 (*p* < 0.001) and sαKL (*p* = 0.001), with higher BMI (SDS) (*p* < 0.001) and WC (SDS) (*p* = 0.003) than controls (Table 1).

**Fig. 1:**
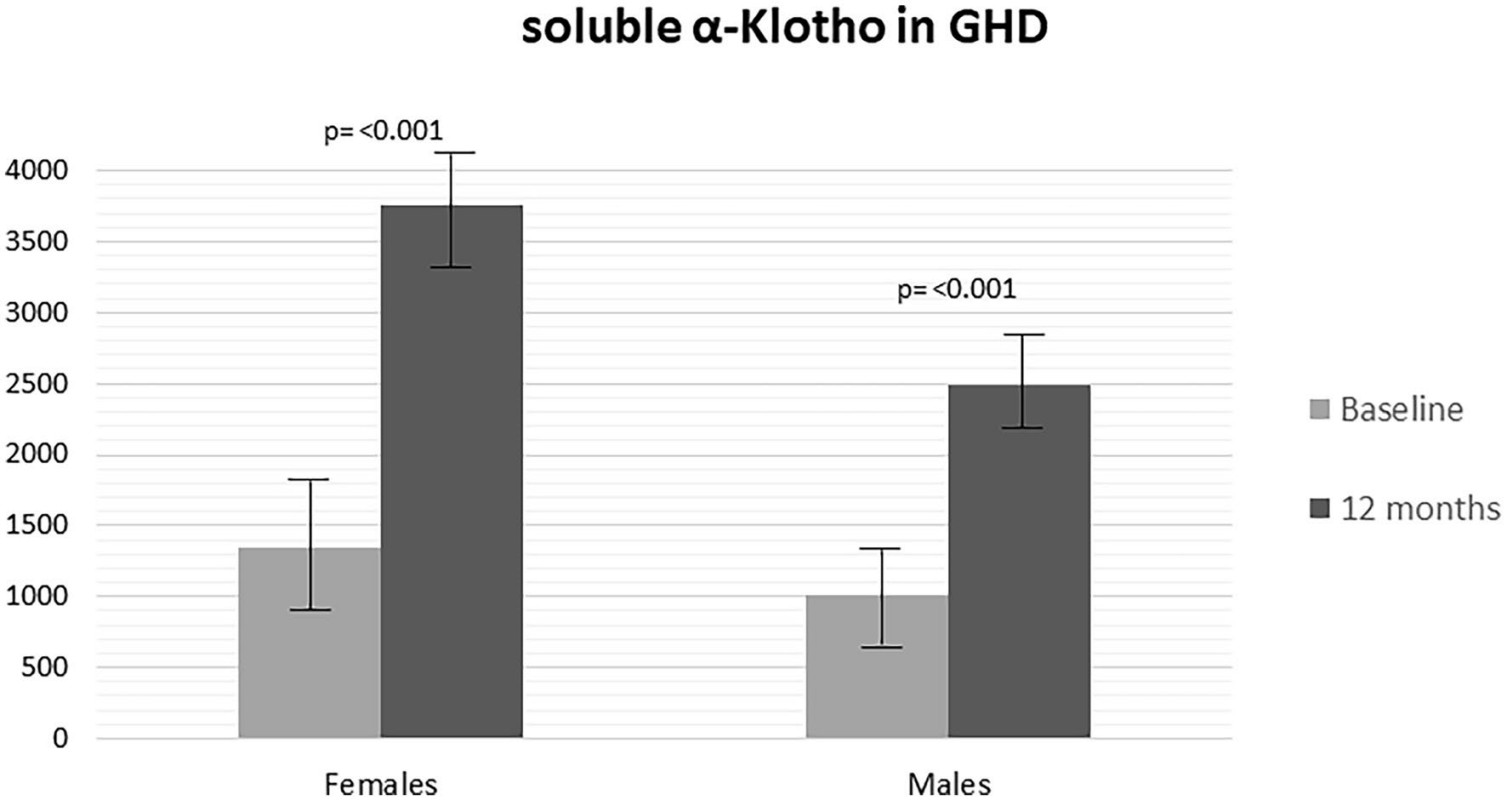
Comparison of sαKL levels between male and female children with GHD at baseline and after 12 months of treatment

After 12 months of follow-up, controls had a significant increase in height (SDS) (*p* < 0.001), BMI (SDS) (*p* = 0.002), WC (SDS) (*p* < 0.001), height velocity (SDS) (*p* < 0.001) and sαKL (*p* = 0.030; females *p* = 0.045 and males *p* = 0.024) (Table 2) than baseline. After 12 months of GHT, GHD children showed a significant increase in height (SDS) (*p* < 0.001), growth velocity (SDS) (*p* < 0.001), bone/chronological age ratio (*p* < 0.001), IGF-1 (*p* < 0.001), fasting insulin (*p* < 0.001), Homa-IR (*p* < 0.001) and sαKL (*p* < 0.001; females *p* = 0.004 and males *p* = 0.001) levels, with a concomitant decrease in BMI (SDS) (*p* = 0.002) and WC (SDS) (*p* = 0.038) compared to baseline (Table 2). A comparison between sαKL levels at baseline and after 12 months of treatment in males and females with GHD was performed, showing that females with GHD had higher sαKL values than males (Fig. 1). No other differences between females and males were observed in controls and GHD (data not shown).

**Table 2:** Clinical, hormonal and metabolic parameters of controls and GHD children at diagnosis and after 12 months

	Controls baseline No = 56	Controls 12 months No = 56	*p*	GHD baseline No = 58	GHD 12 months No = 58	p*	p**
	Mean ± SD	Mean ± SD		Mean ± SD	Mean ± SD		
Height (SDS)	− 1.8 ± 0.5	− 1.41 ± 0.42	< 0.001	− 2.11 ± 0.71	− 1.58 ± 0.91	< 0.001	0.205
BMI (SDS)	− 0.99 ± 0.47	− 0.69 ± 0.37	0.002	− 0.51 ± 0.27	− 0.67 ± 0.37	0.002	0.773
Waist circumference (SDS)	− 0.31 ± 0.15	− 0.12 ± 0.08	< 0.001	0.3 ± 0.1	0.1 ± 0.08	0.038	< 0.001
Height velocity (SDS)	− 0.59 ± 0.19	− 0.23 ± 0.12	< 0.001	− 0.95 ± 0.53	− 0.43 ± 0.05	< 0.001	< 0.001
Bone/chronological age ratio	0.89 ± 0.09	0.91 ± 0.11	0.891	0.76 ± 0.13	0.90 ± 0.09	< 0.001	0.595
IGF-I (µg/L)	100.5 ± 12.5	148.9 ± 21.5	0.624	74.1 ± 27	147.3 ± 37.1	< 0.001	0.061
Fasting glucose (mmol/L)	4.11 ± 0.42	4.08 ± 0.36	0.866	4.32 ± 0.59	4.38 ± 0.36	0.057	< 0.001
Fasting insulin (µU/mL)	4.92 ± 3.10	4.78 ± 2.95	0.805	6.2 ± 3.3	9.1 ± 3.8	< 0.001	< 0.001
HbA1c (%)	5.24 ± 0.29	5.18 ± 0.25	0.538	5.2 ± 0.33	5.3 ± 0.34	0.686	0.053
Homa-IR	1.2 ± 0.75	1.4 ± 0.53	0.112	0.87 ± 0.46	2.02 ± 0.76	< 0.001	< 0.001
ISI-Matsuda	11.6 ± 4.2	10.9 ± 4.8	0.409	12.2 ± 4.5	10.5 ± 2.35	0.065	0.571
Oral disposition Index	0.56 ± 4.21	0.63 ± 3.87	0.926	0.48 ± 4.47	1.01 ± 2.04	0.068	0.128
Total cholesterol (mmol/L)	3.89 ± 0.52	3.63 ± 0.48	0.065	4.14 ± 0.71	3.77 ± 0.64	0.401	0.198
HDL cholesterol (mmol/L)	1.65 ± 0.33	1.66 ± 0.27	0.513	1.61 ± 0.21	1.67 ± 0.24	0.260	0.834
LDL cholesterol (mmol/L)	1.98 ± 0.56	1.86 ± 0.66	0.096	2.02 ± 0.69	1.97 ± 0.71	0.104	0.279
Triglycerides (mmol/L)	1.61 ± 0.51	1.57 ± 0.54	0.685	1.49 ± 0.54	1.53 ± 0.55	0.270	0.696
sαKL (pg/mL) Females Males	1594.5 ± 461.3 1953.4 ± 1024.7 1273.7 ± 430.3	1879.4 ± 567.3 2124.2 ± 1073.2 1583.7 ± 465.8	0.030 0.045 0.024	1136.5 ± 649.9 1607.1 ± 493.5 1034.3 ± 472.8	2776.2 ± 1501.3 3754.2 ± 1834.8 2493.1 ± 1283.4	< 0.001 0.004 0.001	< 0.001 < 0.001 < 0.001

*SDS* standard deviation score, *BMI* body mass index, *WC* waist circumference, *Homa-IR* homeostasis model assessment estimate of insulin resistance, *ISI* insulin sensitivity index, *sαKL* soluble α-klotho

*p* = difference between controls at baseline and after 12 months

p* = difference between GHD children at baseline and after 12 months of GH treatment

p** = difference between controls and GHD children after 12 months

The comparison between GHD children and controls at 12 months showed that GHD children had significantly higher WC (SDS) (*p* < 0.001), fasting glucose (*p* < 0.001), fasting insulin (*p* < 0.001), HOMA-IR (*p* < 0.001), sαKL (*p* < 0.001; females and males *p* < 0.001) levels, than controls (Table 2).

**Fig. 2:**
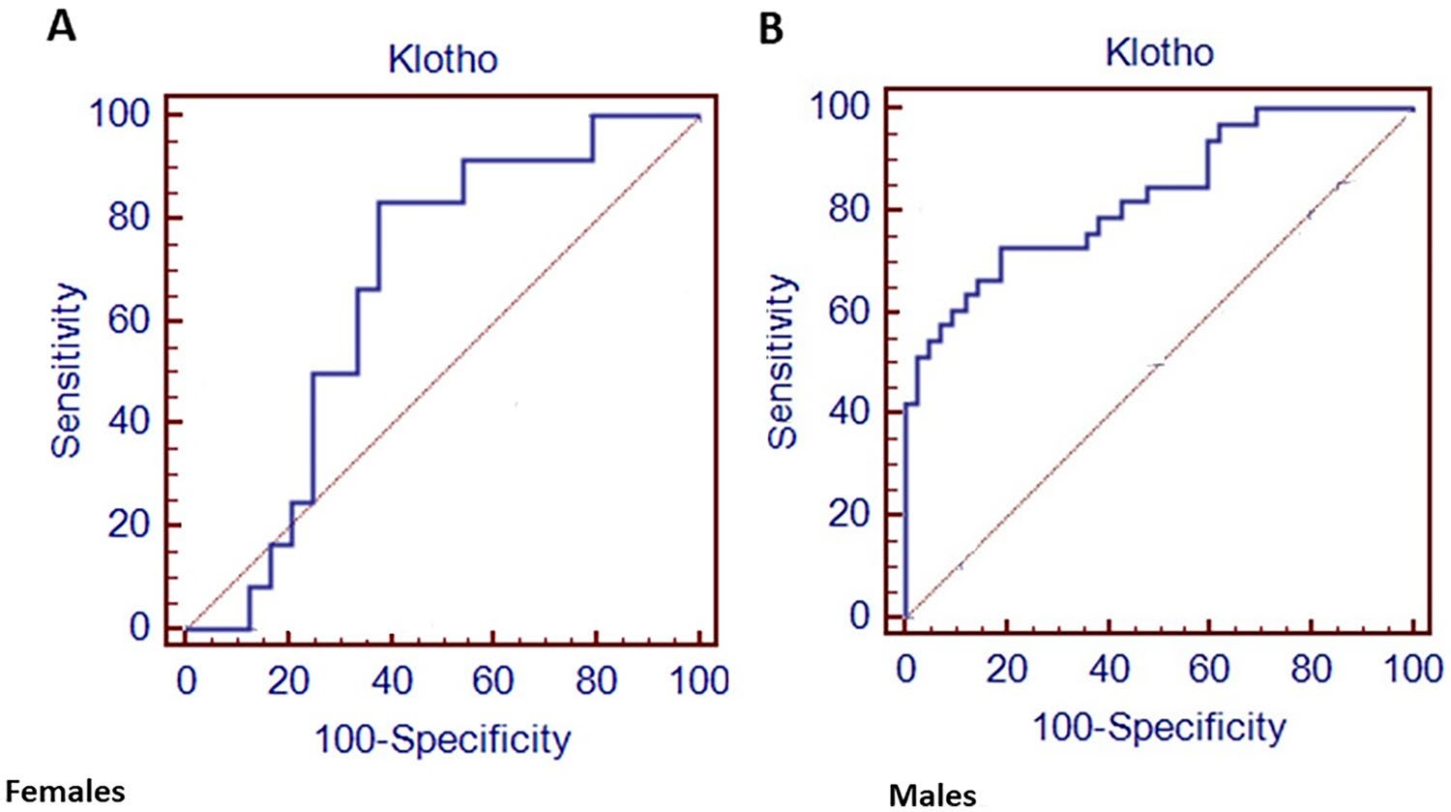
ROC curve analysis of sαKL cut-off in females and males to discriminate controls from GHD

In addition, a ROC curve analysis was performed to identify the sαKL cut-off differentiating GHD children from controls. The sαKL cut-off of 1764.4 pg/mL discriminated female controls and GHD children with 83.3% sensitivity and 62.5% specificity, and the area under the curve was 0.667 (Fig. 2). The sαKL cut-off of 1339.4 pg/mL differentiated male controls and GHD children with a sensitivity of 72.7% and a specificity of 81%; the area under the curve was 0.828 (Fig. 2).

**Fig. 3:**
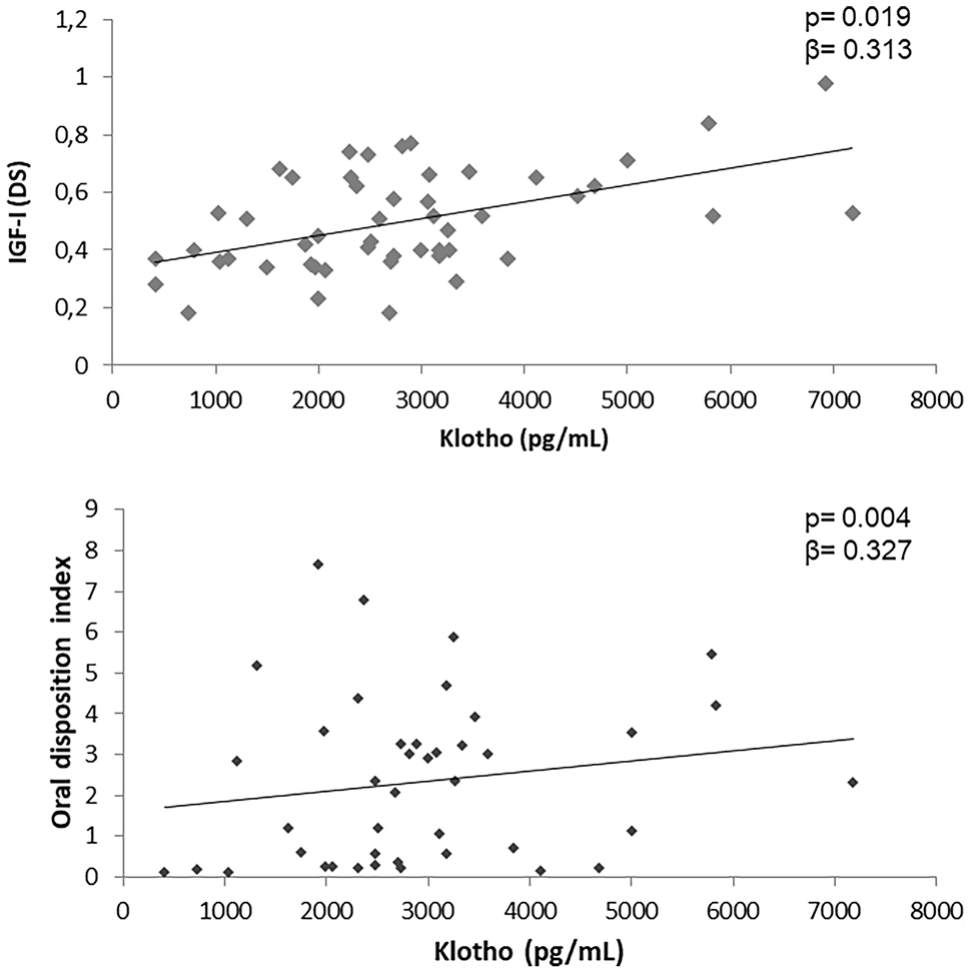
Independent variables associated with sαKL levels after 12 months of GH treatment at multivariate analysis

At multivariate analysis, the independent variables significantly associated with sαKL levels after 12 months of GHT were the oral disposition index (*p* = 0.004, *β* = 0.327) and IGF-1 (*p* = 0.019, *β* = 0.313) (Fig. 3).

## Discussion

In the current study, we evaluated the effects of GHT on sαKL levels in a paediatric cohort of GHD children and healthy controls. As expected from previous reports [30, 31], sαKL was lower at diagnosis in patients with GHD compared to healthy controls and increased after GHT.

Lower sαKL values have been reported in children with GHD compared with controls [30]. Interestingly, patients with organic GHD had lower sαKL levels than idiopathic GHD and GH-sufficient participants [31]. By contrast, some authors did not find differences in sαKL in the diagnosis of GHD compared to short stature, showing superimposable values in the two groups, likely due to the small sample included in the study and maybe to the lack of information on FGF-23 [32].

In the current study we found that the sαKL cut-off to discriminate healthy controls from GHD was 1764.4 pg/mL with a sensitivity of 83.3% and a specificity of 62.5% and 1339.4 pg/mL, with a sensitivity of 72.7% and a specificity of 81% in females and males, respectively.

As expected, IGF-1 was independently associated with sαKL levels. A positive correlation of sαKL and IGF-1 values has been widely demonstrated with interesting close reciprocal regulation [32, 33]. IGF-1 appears to stimulate klotho secretion [30, 34], whereas klotho inhibits IGF-1 signalling and activation of the hormone receptor [19]. A significant increase in IGF-1 and IGFBP3 after intraperitoneal injections of klotho has been reported in mice [35], which seems to contradict the inhibition of klotho on pituitary secretion of GH. However, in GH3-cultured cells it has been reported that klotho induces GH secretion by activation of the ERK1/2 pathway [35]. In these cultured cells cotreatment of klotho and bFGF further increased ERK1/2 phosphorylation, while inhibition of ERK1/2 favours the klotho-induced inhibition of GH release in normal pituitaries [35]. In brief, α-klotho stimulates GH secretion at the expense of ERK1/2 phosphorylation and blocks the inhibitory effect of IGF-1 on GH secretion in GH-secreting adenoma cultures [35].

In addition, we found a gender difference in sαKL. To our knowledge, this is the first time that this has been reported in humans, because till now it has only been shown in animals [36]. However, further studies are required to confirm our results.

Interestingly, we also found that insulin secretion expressed by the oral disposition index, a composite measure of β-cell function, which estimates the ability of β-cells to produce insulin adjusted for insulin sensitivity, was independently associated with sαKL levels. Insulin has been demonstrated to stimulate α-klotho [37, 38]. Recently, an exacerbation and aggravation of insulin resistance in sαKL deficiency was reported in patients with type 2 diabetes mellitus, while overexpression of sαKL was associated with increased insulin sensitivity [39]. On the other side, klotho might induce insulin resistance in adipocytes, preventing insulin effects on promotion of GLUT4 plasma membrane translocation, and attenuating intracellular insulin signalling through main mediators, such as Akt, GSK3β, and PFKf3β [40]. However, detailed information about the relationship between sαKL and insulin and the possible role of rhGH is still lacking and needs to be further investigated.

GHT has been suggested to impair insulin sensitivity, even though many studies have investigated insulin sensitivity by HOMA-IR, a basal index not enough reliable to assess insulin sensitivity [41].

The increase in Homa-IR, may just represent an expected consequence of GH-induced basal hyperinsulinemia and currently very few studies have investigated different indices [8]. In the current study, we found an increase in HOMA-IR, but no changes in ISI Matsuda and DIo were found after 12 months of GHT in GHD children.

A limitation of the study is that we do not have full information about the nutrition and physical activity of the children. The strength of the study is relevant number of children enrolled, with a very homogeneous sample (all children were pre-pubertal).

In conclusion, the findings of this study suggest that sαKL may be used as a marker of GHD combined with IGF-1 and GH. Direct GH measurements alone are useless due to the pulsatile nature of GH secretion, while IGF-1 levels alone are unsatisfactory as well as being influenced by age, gender (oestrogens), race, genetics, liver function, nutritional status, portal insulin, thyroid hormones, and concomitant inflammatory disease [42]. A gender-related cut-off of sαKL to discriminate controls from GHD children was identified. Insulin and IGF-1 are independently associated with sαKL values after 12 months of GHT, supporting the interesting relationship between sαKL levels and insulin/IGF-1 signalling.

However, further larger prospective studies are needed to confirm our results.
